# Metabolic Profiling and Detoxification of Eupalinolide A and B in Human Liver Microsomal Systems

**DOI:** 10.3390/toxics14030235

**Published:** 2026-03-09

**Authors:** Yingzi Li, Xiaoyan Liu, Ludi Li, Wusheng Xiao, Youbo Zhang, Kewu Zeng, Qi Wang

**Affiliations:** 1 Department of Toxicology, School of Public Health, Peking University, Beijing 100191, China; liyingzi@bjmu.edu.cn (Y.L.); yanzi_89@163.com (X.L.); liludi@bjmu.edu.cn (L.L.); wxiao@bjmu.edu.cn (W.X.); 2 Key Laboratory of State Administration of Traditional Chinese Medicine for Compatibility Toxicology, School of Public Health, Peking University, Beijing 100191, China; 3 Key Laboratory of Toxicological Research and Risk Assessment for Food Safety, School of Public Health, Peking University, Beijing 100191, China; 4 State Key Laboratory of Natural and Biomimetic Drugs, School of Pharmaceutical Sciences, Peking University, Beijing 100191, China; ybzhang@bjmu.edu.cn; 5 Department of Integration of Chinese and Western Medicine, School of Basic Medical Sciences, Peking University, Beijing 100191, China

**Keywords:** eupalinolide A, eupalinolide B, metabolite profiling, human liver microsomes, hepatocyte toxicity

## Abstract

Eupalinolide A (EA, *Z*-configuration) and Eupalinolide B (EB, *E*-configuration) are *cis-trans* isomeric sesquiterpenoid monomers isolated from *Eupatorium lindleyanum* DC. (Asteraceae). Although these compounds display anti-inflammatory and anti-tumor activities, their metabolite profiles and possible hepatotoxicity remain largely unknown. This study aimed to investigate the metabolic profiles of EA and EB in liver microsomes and clarify whether they undergo metabolic activation or detoxification. EA and EB were metabolically profiled in human liver microsomes (HLMs) via UPLC-Q-TOF-MS. A HepG2-HLM co-culture system was used to compare the hepatocyte toxicity of parent compounds and their hydrolysis, oxidation, and hydrolysis–oxidation metabolites, thus evaluating their metabolic detoxification pathways. Sixteen metabolites of EA and 19 of EB were identified, with hydrolysis being the predominant metabolic pathway for both isomers. Both compounds showed low hepatocyte toxicity and underwent metabolic detoxification mainly via hydrolytic and oxidative pathways. Notably, hydrolysis metabolites had significantly lower toxicity than oxidative products in HepG2 cells. These results suggest that EA and EB could present a relatively low risk of in vivo hepatotoxicity, which provides useful information for understanding the metabolic behavior and safety profile of these bioactive sesquiterpenoids.

## 1. Introduction

**Figure 1:**
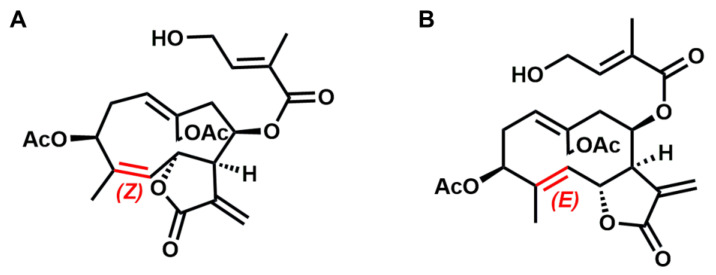
Chemical structures of EA (**A**) and EB (**B**). The stereochemical configurations of the double bond are highlighted in red: Z for EA and E for EB.

Drug-induced liver injury (DILI), a major safety concern in pharmacotherapy, is the predominant cause of clinical trial early-termination and post-marketing drug withdrawals [1,2]. Consequently, characterizing DILI-associated metabolites is critical for pharmaceutical safety during drug development [3]. Eupalinolide A (EA, *Z*-configuration) and Eupalinolide B (EB, *E*-configuration), a pair of *cis-trans* isomeric sesquiterpenoids (Figure 1), were isolated from *Eupatorium lindleyanum* DC., a medicinal plant of the Asteraceae family. They have been reported to possess anti-inflammatory and anti-tumor properties in preclinical studies [4,5,6,7]. EB has emerged as a promising anti-neuroinflammatory candidate for treating neurodegenerative diseases through its selective binding to ubiquitin-specific protease 7 [8]. Recently, EB was found to promote K48-linked polyubiquitination and proteasomal degradation of DEK proto-oncogene protein (DEK) at Lys349 via ring finger protein 149 (RNF149) and RNF170, thereby suppressing the DEK-receptor-interacting protein kinase 1 (RIPK1)-PANoptosis axis to alleviate airway inflammation and eosinophil infiltration in allergic asthma [9]. Although EA and EB possess promising therapeutic potential, studies on their metabolic profiles and toxicity remain scarce to date. A pharmacokinetic study in rats demonstrated distinct metabolic parameters between these isomers, with EB exhibiting higher bioavailability and thus greater clinical potential than EA [10]. Recently, we reported that EA and EB were rapidly metabolized primarily through carboxylesterase-mediated hydrolysis. Additionally, CYP3A4 was identified as the principal cytochrome P450 isoform responsible for their oxidation, which exhibited notable stereoselectivity in metabolism [11]. However, evidence regarding the metabolite profiles and toxicity characteristics of EA and EB remains limited, which is indispensable for further preclinical evaluation before clinical trials [12]. Furthermore, existing studies have not established a direct correlation between the metabolic processes of EA/EB and their hepatotoxicity, nor have they clarified the differential effects of metabolic pathways, such as hydrolysis and oxidation, on the detoxification or metabolic activation of these compounds.

We hypothesized that EA and EB undergo metabolic detoxification and that hydrolysis is the major pathway that reduces toxicity, while oxidative metabolites remain more toxic than hydrolytic ones. As the liver is the primary metabolic organ for orally administered drugs, we used human liver microsomes (HLMs), a classic system for predicting human metabolic profiles in preclinical investigation. Meanwhile, ultra-performance liquid chromatography coupled with quadrupole time-of-flight mass spectrometry (UPLC-Q-TOF-MS) was applied to identify metabolites [13,14], and quantitative structure–activity relationship (QSAR) methods were used to predict potential toxicity. Hepatotoxicity was further validated using a HepG2-HLM co-culture system. This study first elucidated the hydrolysis-centered metabolic detoxification mechanism of EA and EB, and clarified the structure–activity relationship, showing that hydrolytic metabolites exhibit significantly lower toxicity than oxidative metabolites. These findings fill the knowledge gap in the metabolism–toxicity relationship of EA/EB and provide experimental support for their safety evaluation.

## 2. Materials and Methods

### 2.1. Reagents and Materials

EA (purity ≥ 98% by HPLC) was obtained from Beijing Mreda Technology Co., Ltd. (Beijing, China), and EB (purity ≥ 98% by HPLC) was purchased from Chengdu Push Biotechnology Co., Ltd. (Chengdu, China). Carbamazepine was purchased from Shanghai Yuanye Biotechnology Co., Ltd. (Shanghai, China). Alamethicin was purchased from Meilun Cloud Cell Biotechnology Co., Ltd. (Beijing, China). Ethylenediamine tetraacetic acid (EDTA) and dimethyl sulfoxide (DMSO) were obtained from Amresco (Solon, OH, USA). Nicotinamide adenine dinucleotide phosphate (NADPH) and uridine diphosphate glucuronic acid (UDPGA) were purchased from Sigma Aldrich (St. Louis, MO, USA). Acetonitrile and methanol for high-performance liquid chromatography (HPLC) and mass spectrometry (MS) were obtained from Thermo Fisher Scientific (Waltham, MA, USA). Pooled HLMs (20 g/L) were purchased from Xeno Tech (Kansas City, KS, USA); HLM donors were individuals without hepatic disease and included 100 men and 100 women aged 16–78 years (Lot no. 1910096); the supplier has obtained the necessary informed consent and ethical approval for tissue collection (Supplementary Material S2). The activity of HLMs was confirmed via the detection of major metabolic enzymes (CYP1A2, CYP2C9, CYP2C19, CYP2D6, CYP3A4, UGT1A1, UGT1A3, UGT1A4, UGT1A9, and UGT2B7). Ultra-pure water was purified by a Milli-Q system (Millipore, Billerica, MA, USA). All other chemicals and reagents were of analytical grade and commercially available.

The HepG2 cell line was obtained from the National Biomedical Experimental Cell Resource Bank. Dulbecco’s modified Eagle’s medium (DMEM) was obtained from Procell Life Science &Technology Co., Ltd. (Wuhan, China). Fetal bovine serum (FBS) and trypsin-EDTA (0.25%) were purchased from Gibco™ Laboratories (Life Technologies Inc., Grand Island, NY, USA). Penicillin-streptomycin solution (100×) was supplied by Biological Industries (Kibbutz Beit Haemek, Israel). Cell culture grade DMSO was purchased from Meilun Biotech Co., Ltd. (Dalian, China). Enhanced Cell Counting Kit-8 (CCK-8) and phosphate-buffered solution (PBS) were supplied by Beyotime Biotechnology Co., Ltd. (Shanghai, China). Bis(4-nitrophenyl) phosphoric acid (BNPP) was purchased from Beijing Innochem Technology Co., Ltd. (Beijing, China).

### 2.2. Metabolite Profiling

#### 2.2.1. HLMs Incubation System and Sample Pretreatment

The incubation solution contained pooled HLMs (0.5 g/L, resuspended microsomal pellet), EDTA (1 mM), alamethicin (25 mg/L), MgCl_2_ (5 mM), NADPH (1 mM), and UDPGA (5 mM), in a total volume of 200 μL in 100 mM potassium phosphate buffer, pH 7.4 [15,16,17,18]. The mixture was placed on ice for 15 min, and then pre-incubated at 37 °C for 5 min. Reactions were initiated with the addition of EA or EB (10 µM of each). Such substrate concentration was used based on previous pharmacokinetic data showing that the peak plasma concentration (C_max_) of EB in rats after oral administration of 50 mg/kg was approximately 1.2 μM [8]. Thus, the 10 µM concentration used herein is a reasonable 8~10-fold magnification, which aligns with the conventional design of in vitro metabolism studies to ensure detectable metabolite levels without exceeding the linear range of enzymatic reactions. The reactions were terminated after 1 h incubation by adding 200 µL ice-cold acetonitrile/methanol (1:1) containing IS (carbamazepine). This duration was justified by our previous metabolic stability data showing that EA and EB exhibit rapid clearance in HLMs with half-lives (*t*_1/2_) of 21.37 min and 21.93 min, respectively [11]. An hour incubation ensures complete metabolic turnover of the parent compounds (≥95% clearance) while avoiding non-physiological metabolite accumulation, consistent with the in vivo hepatic metabolic timeline of short-half-life compounds. The samples were then vortexed for 1 min, vibrated by ultrasonicator for 5 min, and centrifuged for 15 min (17,000 *g*, 4 °C). The supernatants were collected and subjected to evaporation at 45 °C until dry using a vacuum centrifugal concentrator. The samples were resuspended in 200 µL methanol. After vortex, ultrasonic shaking and centrifugation as above, the supernatants were subjected to analysis by UPLC-Q-TOF-MS. The organic solvent content in the incubation system did not exceed 0.5%.

#### 2.2.2. UPLC-Q-TOF-MS Analysis of EA and EB Metabolites

The metabolites of EA and EB in HLMs were analyzed by UPLC-Q-TOF-MS equipped with a LC-30AD binary pump, an SIL-30AC autosampler, an SPD M30A PDA detector, and a CTO-20AC thermostated column compartment. The samples were separated on an ACQUITY UPLC^®^ BEH Shield RP-C18 VanGuardTM column (100 mm × 2.1 mm, 1.7 μm, Waters, Milford, MA, USA) with an ACQUITY UPLC^®^ BEH Shield RP-C18 VanGuardTM precolumn (5 mm × 2.1 mm, 1.7 μm, Waters, Milford, MA, USA). The mobile phase consisted of H_2_O (A) and acetonitrile (B). A gradient elution procedure was optimized as follows: 0~4 min, 5~30% B; 4~8 min, 30~60% B; 8~12 min, 60~98% B; 12~15 min, 98% B; 15~15.1 min, 98~5% B; 15.1~20 min, 5% B. The autosampler temperature and column temperature were 4 °C and 30 °C, respectively. The flow rate was maintained at 0.2 mL/min, and the injection volume was 2 μL. The instrument was set in the electrospray ionization positive and negative ion data-dependent acquisition (DDA) mode with the major parameters listed in Table S1. Carbamazepine was selected as the internal standard to account for analytical variability due to its chemical stability, distinct retention time relative to EA and EB, and consistent, reproducible detector response.

#### 2.2.3. Metabolite Identification

The 2D molecular structure files (mol) of EA and EB, generated using ChemDraw Professional 16.0, along with the corresponding MS/MS spectra, were imported into MetabolitePilot to systematically identify all potential metabolites derived from the EA and EB core structure. According to the Schymanski scale [19], metabolite identification was performed based on MS/MS data without authentic standards, and all annotated metabolites were assigned at Level 2 or Level 3 confidence.

### 2.3. Hepatotoxicity Prediction of EA, EB, and Their Metabolites by QSAR Models

ChemDraw Professional 16.0 was used to generate 2D structural mol files of the metabolites derived from EA and EB. The acute toxicity and hepatotoxicity of EA and EB were predicted using ADMET Predictor 8.5 software (Shanghai VMO Information Technology Co., Ltd., Shanghai, China). Additionally, hepatotoxicity predictions for EA, EB, and their metabolites were performed using the DL-Liew model in the DL-DILI Prediction Server (http://repharma.pku.edu.cn/DILIserver/DILIhome.php, accessed on 19 July 2023), developed by Peking University [20]. The model maintained robust predictive capability in the valRANDOM external validation cohort (120 compounds) with 75.0% accuracy, 81.9% sensitivity, and 64.6% specificity [20].

### 2.4. Hepatocyte Toxicity Testing

#### 2.4.1. Cell Culture

HepG2 cells were maintained in a CO_2_ incubator (37 °C, 5% CO_2_/95% air environment, 95% humidity) and cultured in DMEM supplemented with heat-inactivated FBS (10%), penicillin (100 IU/mL) and streptomycin (100 µg/mL). Cells were sub-cultured every 3~4 days (roughly 80% confluency) with 0.25% trypsin/EDTA.

#### 2.4.2. Hepatocyte Toxicity Changes in EA/EB in Different Metabolic Patterns

The HepG2-HLM co-culture model [21] was adopted with slight modifications to compare the hepatocyte toxicity of EA, EB, and their metabolites. Based on our previous findings demonstrating the rapid metabolic clearance of both EA (*t*_1/2_ = 21.37 min) and EB (*t*_1/2_ = 21.93 min) [11], we employed a 4-h exposure protocol. The HepG2-HLM co-culture system was designed to specifically evaluate the acute hepatocellular toxicity of EA/EB and their metabolic derivatives. The experimental groups were as follows:

(1) Prototype groups: solvent control groups: 0.4% DMSO; treatment groups: EA or EB (12.5, 25, 50, 100 µM).
(2) Hydrolysis–oxidation metabolite groups: solvent control groups: 0.4% DMSO + 0.16 g/L HLMs + 0.5 mM NADPH; treatment groups: EA or EB (12.5, 25, 50, 100 µM) + 0.16 g/L HLMs + 0.5 mM NADPH.
(3) Hydrolysis metabolite groups (without NADPH, which is a coenzyme for initiating phase I reaction): solvent control groups: 0.4% DMSO + 0.16 g/L HLMs; treatment groups: EA or EB (12.5, 25, 50, 100 µM) + 0.16 g/L HLMs.
(4) Oxidation metabolite groups (with BNPP, which is a carboxylesterase inhibitor [22]): solvent control groups: 0.4% DMSO + 0.16 g/L HLMs + 0.5 mM NADPH + 100 µM BNPP; treatment groups: EA or EB (12.5, 25, 50, 100 µM) + 0.16 g/L HLMs + 0.5 mM NADPH + 100 µM BNPP.

HepG2 cells were seeded at a density of 1.5 × 10^5^ cells/mL in a 96-well plate. After 24 h, the medium was removed and replaced with 100 μL of the above treatment solution at various concentrations. After incubation at 37 °C for 4 h, the treatment solution was discarded and replaced with 100 μL CCK-8 solution (10%, diluted with PBS), and incubated at 37 °C for 20 min. Quantitation was performed with the SPECTROstar Nano microplate reader (Ortenberg, Germany) at 450 nm and 650 nm as reference wavelengths. Experiments were performed in triplicate. Relative cell viability (%) was calculated as follows:

OD_treatment/blank/vehicle_ = OD_450_ − OD_650_

Relative cell viability (%) = [(OD_treatment_ − OD_blank_)/(OD_vehicle_ − OD_blank_)] × 100%

The half maximal inhibitory concentration (IC_50_) was determined as the concentration required to achieve 50% inhibition of cell viability, and was calculated with a four-parameter logistic model using GraphPad Prism 9, where curve fitting and graphical representation were also performed.

### 2.5. Statistical Analyses

Statistical analyses were performed using SPSS Statistics 26.0 (IBM, USA) for data processing and GraphPad Prism 9 (San Diego, CA, USA) for graphical representation. All quantitative data are presented as mean ± standard deviation. The Shapiro–Wilk normality test was used to examine the normality of all data. All data were normally distributed, and the homogeneity of variances was verified using Levene’s test. One-way ANOVA followed by Dunnett’s *t*-test was used for multiple comparisons. A *p* < 0.05 was considered statistically significant.

## 3. Results

### 3.1. Metabolite Profiling of EA and EB

**Figure 2:**
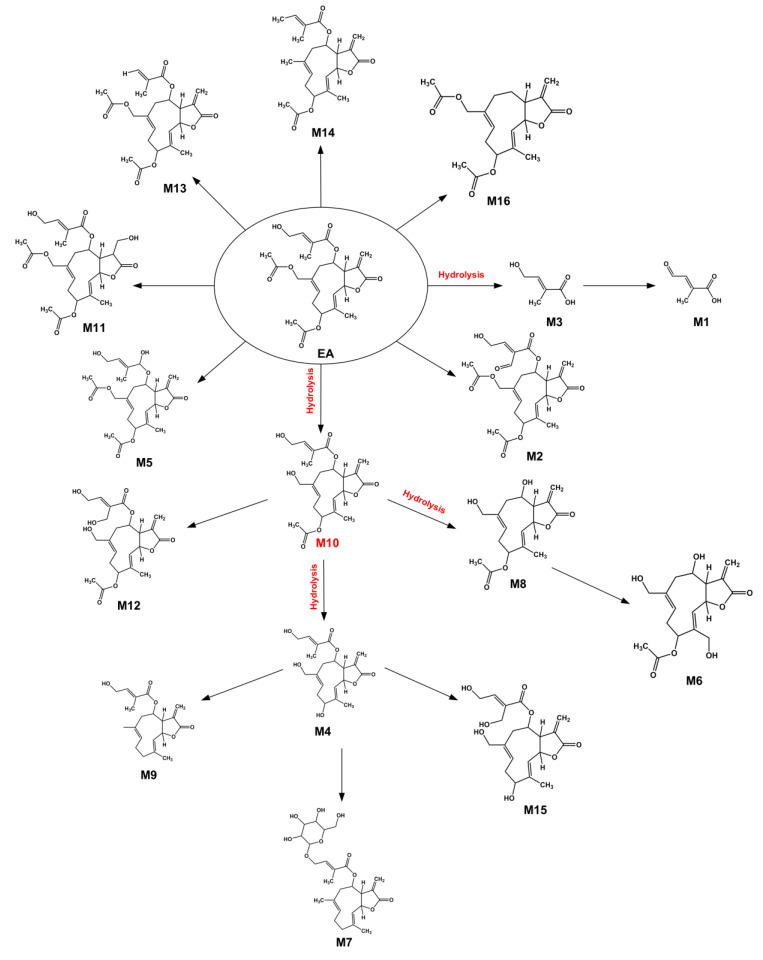
Proposed metabolic pathways of EA in HLMs.

Using MetabolitePilot for metabolite prediction followed by the structural annotation with diagnostic fragment ions obtained from MS/MS analysis (Figure S1–S4), we identified 16 metabolites of EA. These were products of phase I metabolic reactions, including oxidation, double bond reduction, ester bond hydrolysis, ketone formation, demethylation, and deoxygenation, and phase II metabolic reactions, such as glucoside conjugation (Table 1 and Figure 2). Ten of these metabolites (M1, M3, M4, M6, M7, M8, M9, M10, M12, and M15) were associated with hydrolysis reactions, which were related to the ester bond hydrolysis (Table 1 and Figure 2). The hydrolysis of 3 ester bonds in the side chain groups was mainly involved, while the lactones were unchanged (Table 1 and Figure 2). Therefore, the main metabolic reaction of EA was confirmed to be hydrolysis.

**Figure 3:**
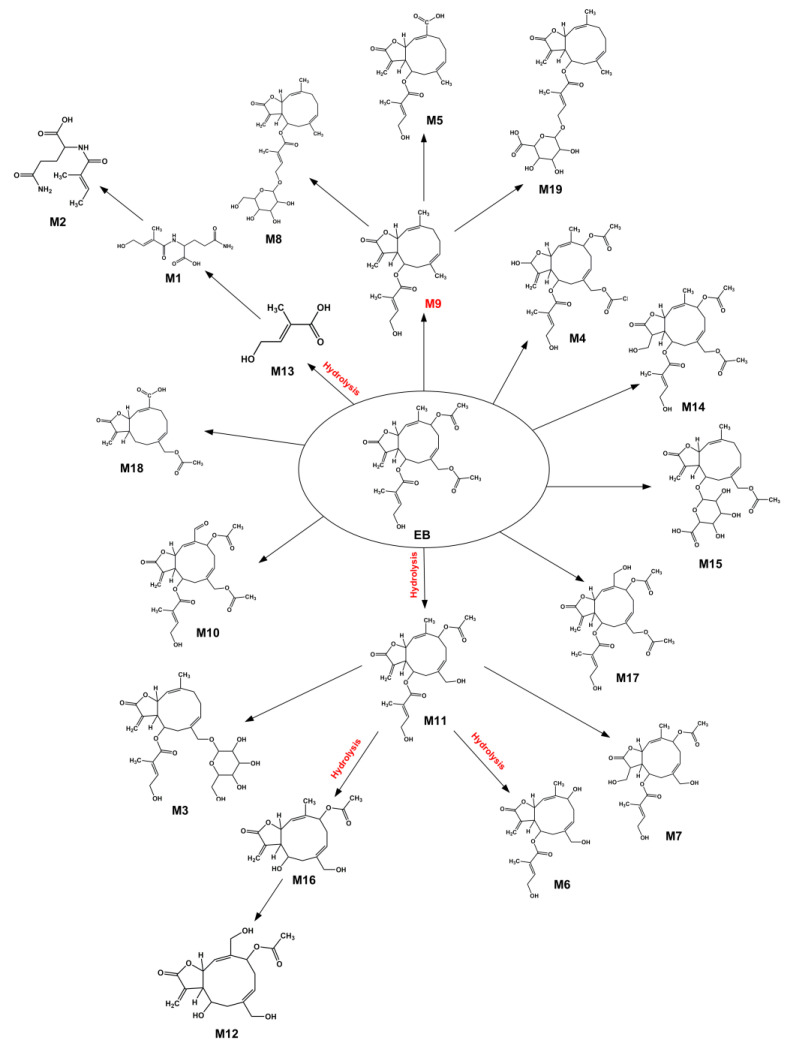
Proposed metabolic pathways of EB in HLMs.

Similarly, a total of 19 metabolites of EB were identified and produced via phase I metabolic reactions (i.e., oxidation, double bond reduction, ester bond hydrolysis, ketone formation, and demethylation) and phase II metabolic reactions (i.e., glutamine conjugation, glucuronic acid conjugation, and glucoside conjugation) (Table 2 and Figure 3). There were 9 metabolites of EB associated with the hydrolysis reaction (M1, M2, M3, M6, M7, M11, M12, M13, and M16), which were related to the hydrolysis of ester bonds (Table 2 and Figure 3). Similar to EA, the hydrolysis mainly involved the 3 ester bonds of side chain groups in the absence of the lactones. Therefore, in line with EA, the main metabolic reaction of EB was also confirmed to be hydrolysis.

### 3.2. Toxicity Prediction of EA, EB, and Their Metabolites by QSAR Model

We next predicted the potential hepatotoxicity of EA, EB, and their metabolites using computational methods. Results from the ADMET Predictor 8.5 indicated that both EA and EB exhibited low or negligible acute toxicity and no hepatotoxicity (Table 3). However, the DL-DILI Prediction Server predicted that both compounds and their metabolites were hepatotoxic (Table 4 and Table 5). Specifically, the phase I metabolites (M1~6, M8~11, and M14~16) of EA formed by oxidation, double bond reduction, hydrolysis, ketone formation, and deoxygenation were predicted as “positive” hepatotoxicity. Only M12 (the product of hydrolysis and oxidation reactions) and M13 (the product of demethylation reaction) were predicted as “negative” hepatotoxicity. Moreover, the phase II metabolites (M7) generated by glucose conjugation were predicted to be nontoxic to the liver.

Similarly, the majority of phase I metabolites of EB (M4~6, M9~13, M14, and M16~17) were predicted to be “positive” hepatotoxicity, including products of oxidation, double bond reduction, hydrolysis, ketone formation, and demethylation (Table 5). Only M7 (the product of ester bond and internal hydrolysis) and M18 (the product of hydrolysis and demethylation) were predicted as “negative” hepatotoxicity. In contrast to EA, most of the phase II metabolites (M1, M3, M8, M15, and M19), which were the products of glutamine conjugation, glucose conjugation, and glucuronidation, were predicted as “positive” hepatotoxicity (Table 5). Only M2 (the product of hydrolysis and glutamine conjugation) was predicted as “negative” hepatotoxicity (Table 5).

The inconsistent predictions between ADMET Predictor 8.5 and DL-DILI Prediction Server could stem mainly from their distinct mechanisms and training sets. ADMET Predictor 8.5 considers metabolic detoxification and gives low hepatotoxicity, while DL-DILI overpredicts toxicity by focusing only on structural alerts without incorporating metabolic disposition.

### 3.3. Metabolic Detoxification of EA and EB

**Figure 4:**
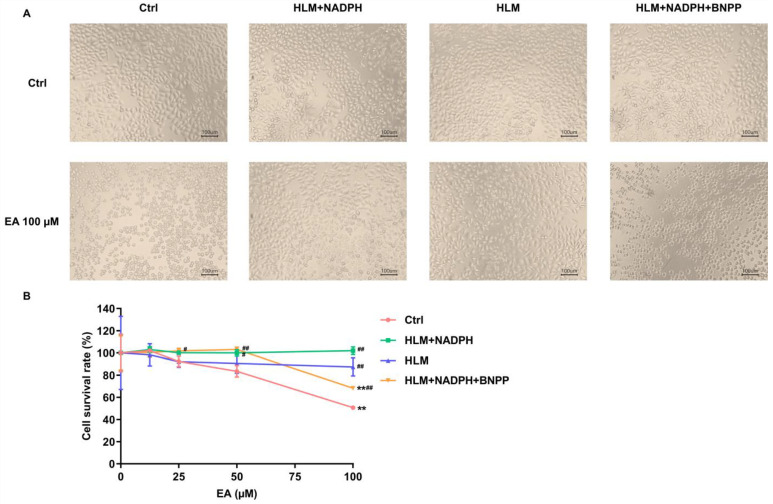
Effects of EA and its metabolites on cell survival of HepG2 cells. (**A**) The HepG2 cells morphology after treatment of 100 µM EA with or without HLMs/NADPH/BNPP for 4 h. (**B**) The viability of HepG2 cells treated with EA in the presence or absence of HLMs/NADPH/BNPP for 4 h ($\bar{x}\;$ ± *s*, *n* = 3). ** *p* < 0.01 vs. vehicle control of the same treatment; ^#^ *p* < 0.05 vs. prototype of the same concentration; ^##^ *p* < 0.01 vs. prototype of the same concentration.

**Figure 5:**
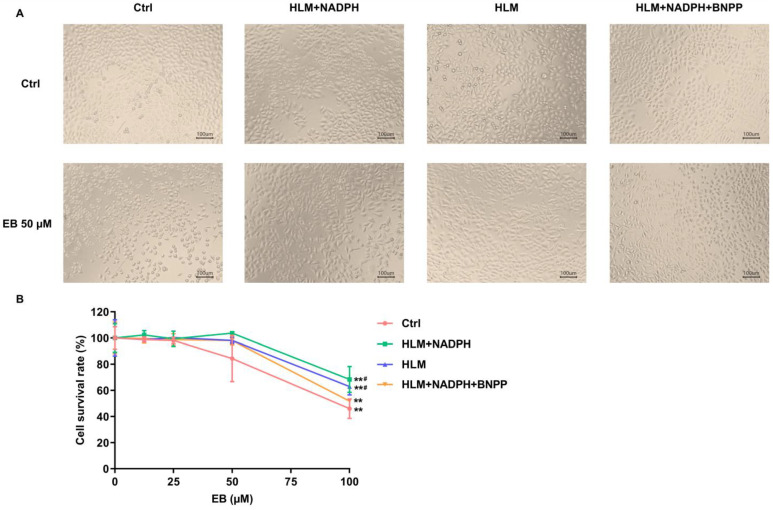
Effects of EB and its metabolites on cell survival of HepG2 cells. (**A**) The HepG2 cells morphology after treatment of 50 µM EB with or without HLMs/NADPH/BNPP for 4 h. (**B**) The viability of HepG2 cells exposed to EB with or without HLMs/NADPH/BNPP for 4 h ($\bar{x}\;$ ± *s*, *n* = 3). ** *p* < 0.01 vs. vehicle control of the same treatment; ^#^ *p* < 0.05 vs. prototype of the same concentration.

Since the prediction results for hepatotoxicity of EA and EB showed discrepancies by different computational models, we further characterized and compared the potential effects of these two compounds using HLMs and HLM-HepG2 cell coculture systems. Due to the limited availability of isolated and enriched metabolite monomers for cellular experiments, this study investigated the parent compounds and enzyme-facilitated metabolic reactions instead. The EA or EB treatment alone groups examined the effects of these two monomeric compounds; the (EA/EB + HLMs + NADPH) groups showed the effects of EA or EB hydrolysis–oxidation metabolites; the (EA/EB + HLMs) groups tested the effects of EA or EB hydrolysis metabolites; and the (EA/EB + HLMs + NADPH + BNPP) groups showed the effects of EA or EB oxidation metabolites. After 4 h treatment with EA/EB in the presence or absence of HLMs/NADPH/BNPP, the HepG2 cell morphology and viability of each group were compared as shown in Figure 4 and Figure 5. For EA or EB treatment, the changes in cell morphology were most pronounced at 100 μM and 50 μM, respectively. Therefore, the above two concentrations were selected to compare cell morphology after treatment with EA or EB.

As shown in Figure 4, in the EA alone treatment and (EA + HLMs + NADPH + BNPP) group at 100 μM, HepG2 cells showed shrinkage and rounded morphology at 4 h, while most of the cell morphology in the (EA + HLMs + NADPH) group and (EA + HLMs) group did not significantly change. The survival rate of HepG2 cells decreased significantly when the EA concentration reached 100 µM in the EA alone treatment and the (EA + HLMs + NADPH + BNPP) group, while the hydrolysis–oxidation and hydrolysis metabolites of EA had no significant effects in the dose range of 0~100 µM. The cell survival rates in (EA + HLMs + NADPH), (EA + HLMs), and (EA + HLMs + NADPH + BNPP) groups were significantly increased compared to those of the EA alone treatment at 100 µM.

As shown in Figure 5, after treatment with 50 µM EB alone for 4 h, the HepG2 cells shrank and became rounded, while the cell morphology of the other three treatment groups did not markedly change. The cell survival rates of all groups decreased significantly when the EB concentration reached 100 µM. The cell survival rates of the hydrolysis–oxidation and hydrolysis metabolites groups were obviously improved compared with those of the EB alone treatment at 50 µM.

The IC_50_ values of HepG2 cells treated by EA or EB with or without HLMs/NADPH/BNPP for 4 h were shown in Table 6. Dose-response curves and corresponding IC_50_ values for EA and EB in HepG2 cells under different incubation conditions were shown in Figure S5. The order of IC_50_ for EA treatment groups was: IC_50_ (EA) < IC_50_ (EA oxidation metabolites). Neither the hydrolysis–oxidation nor the hydrolysis metabolites of EA displayed 50% cellular growth inhibition within the tested concentration range (0~100 μM); thus, the precise IC_50_ values could not be determined. The order of IC_50_ for EB treatment groups was: IC_50_ (EB) < IC_50_ (EB oxidation metabolites) < IC_50_ (EB hydrolysis metabolites) < IC_50_ (EB hydrolysis–oxidation metabolites). These results support the idea that the metabolism of EA or EB is a detoxification process.

## 4. Discussion

We performed the first comprehensive metabolite profiling analysis of EA and EB in HLMs using UPLC-Q-TOF-MS and identified 16 metabolites of EA and 19 metabolites of EB. Structural elucidation demonstrated that hydrolysis served as the principal metabolic pathway for both compounds, accounting for 10 of the 16 EA metabolites and 9 of the 19 EB metabolites. This predominance of hydrolysis metabolites aligns with the structural presence of 3 ester bonds in their respective side-chain moieties. Together with our previous metabolic mechanistic investigations [11], these findings support the idea that the rapid elimination of EA and EB results from carboxylesterase-mediated hydrolysis.

Beyond EA and EB, Qin et al. systematically profiled the in vivo metabolism of three structurally different constituents of *Eupatorium lindleyanum* DC.—eupalinilide B [14], eupalinilide C [13], and eupalinolide F [23]—in rats via high-resolution UPLC-Q-TOF-MS. This study identified 26, 29, and 55 metabolites for eupalinilide B, eupalinilide C, and eupalinolide F, respectively, with hydrolysis emerging as the predominant metabolic pathway for all three compounds. Notably, the molecular structures of EA and EB demonstrated significantly greater instability compared to the above three sesquiterpene lactones, attributable to their distinctive chemical architecture featuring three ester bonds and one lactone moiety. However, our results revealed fewer metabolites derived from EA and EB relative to the aforementioned three compounds. This discrepancy may reflect: (1) fundamental differences between in vitro and in vivo metabolic conditions, with the latter being a more complex biotransformation environment and broader enzyme repertoire; and (2) potential interspecies variations in drug metabolism between rodents and humans [24].

The pooled HLMs used in this study were obtained from 200 healthy donors without known liver diseases. This design reduces individual differences by integrating metabolic enzyme activities from a diverse population. Our results reflect the metabolic characteristics of the general healthy population, which meet the requirements of preclinical safety evaluation. HLM incubation conditions were designed in accordance with the classic methodological specifications for in vitro drug metabolism studies [15,16,17,18] and optimized based on the metabolic characteristics of EA and EB. The potential impacts of these conditions and the rationality of their selection are as follows. First, the incubation concentration of EA and EB was set at 10 μM mainly based on two considerations: (1) this concentration ensures sufficient detection sensitivity for metabolites in UPLC-Q-TOF-MS analysis and avoids missing low-abundance metabolites; (2) our previous studies have demonstrated that 10 μM EA/EB exhibits linear metabolism within 30 min in a 200 μL incubation system containing 0.5 g/L HLMs without enzyme saturation, thus preventing deviations in metabolite production. Second, alamethicin functions to increase the permeability of microsomal membranes and facilitate the reaction between cofactors (e.g., NADPH, UDPGA) and metabolic enzymes. The addition of 25 mg/L alamethicin in this study was performed with reference to the routine operating procedures for HLM metabolism studies [15,18]. Third, the simultaneous addition of phase I (NADPH) and phase II (UDPGA) cofactors in the incubation system can simulate the sequential metabolic processes in the liver—intermediates generated from phase I metabolism (oxidation, hydrolysis) immediately undergo phase II conjugation reactions (glucuronidation, etc.), which is consistent with the actual metabolic pathways of drugs in vivo. The addition of only one type of cofactor may lead to the accumulation of intermediate metabolites and fail to reflect the complete metabolic network. Although the simultaneous addition of the two types of cofactors may result in metabolic pathway competition, this is an inherent characteristic of in vivo metabolism. Fourth, even with 5 mM UDPGA, few glucuronide conjugates were detected (none for EA, two for EB), confirming hydrolysis was the predominant pathway, catalyzed efficiently by carboxylesterase in vitro liver microsomal system.

We employed two QSAR models to predict the hepatotoxic potential of EA, EB, and their metabolites. ADMET Predictor 8.5 indicated minimal acute toxicity and negligible hepatotoxicity for both EA and EB, and this prediction was consistent with subsequent in vitro cytotoxicity assays showing low cytotoxicity of EA and EB in HepG2 cells. In contrast to these findings, the DL-DILI Prediction Server predicted that EA, EB, most of their phase I metabolites, and the majority of EB’s phase II metabolites possess hepatotoxic potential. First, the discrepancies in predictions between the two models could be attributed to the fundamental difference in their predictive mechanisms. Specifically, ADMET Predictor 8.5 predicts hepatotoxicity based on QSAR models established with direct cytotoxicity endpoints (e.g., serum AST/ALT/LDH levels) and integrates metabolic stability data for compounds (https://www.simulations-plus.com/software/admet-predictor/ accessed on 6 December 2022). This model recognizes that the three ester bonds in EA and EB are rapidly hydrolyzed by carboxylesterases, leading to minimal accumulation of the parent compounds in hepatocytes. Additionally, the lactone moiety of EA and EB is a saturated lactone that cannot form reactive electrophilic intermediates capable of covalently bound to hepatic macromolecules—a key mechanism underlying lactone-induced hepatotoxicity [25]. Based on these structural and metabolic characteristics, ADMET Predictor 8.5 predicted the absence of hepatotoxicity for EA and EB. In contrast, the DL-DILI Prediction Server is a machine learning ensemble model based on mixed algorithms and structural feature mining [19], which prioritizes the identification of structural moiety associated with indirect hepatotoxicity or clinical DILI, rather than features linked to direct cytotoxicity or metabolic disposition. This model lacks the integration of data on metabolic enzyme-mediated detoxification pathways, resulting in the overprediction of hepatotoxicity for EA, EB, and their phase II conjugates. Second, the DL-DILI Prediction Server predicted hepatotoxicity for most metabolites (including numerous phase II conjugates), which contradicts the conventional detoxification paradigm. The reason may be that phase II conjugations do not eliminate the hepatotoxicity-associated structural motifs, and the introduced polar groups (e.g., glucosyl groups) fail to mask the toxic structural features recognized by the model [26]. Thus, the predictions can only serve as preliminary risk alerts and require experimental validation. Fourth, structural alert analysis for hepatotoxicity revealed that the core structures of EA and EB consist of a sesquiterpene hydrocarbon backbone, one saturated lactone ring, and three side-chain ester bonds, with no classic hepatotoxic structural alerts present. Specifically, the lactone ring is a saturated lactone without α,β-unsaturated double bonds—α,β-unsaturated lactone being the most classic hepatotoxic structural alert in terpenoid lactones—as this structure can form electrophilic intermediates via Michael addition [27]. The three side-chain ester bonds are hydrolysable ester groups that do not act as toxic structural alerts; instead, they enable metabolic detoxification mediated by carboxylesterases. Phase I metabolism of EA and EB, which is predominantly hydrolysis, weakens or eliminates the potential weak structural features of the parent backbone through structural modification without generating new structural alerts, reflecting the preliminary detoxification effect of phase I metabolism. Phase II metabolism of EA and EB involves conjugation with polar groups, which completely ablates all potential toxicity-associated structural features with no de novo generation of structural alerts, representing the core structural mechanism underlying their metabolic detoxification.

Consistent with conventional toxicity classification criteria, the observed IC_50_ values > 50 μM in hepatocyte assays indicate negligible toxicity under these experimental conditions [28]. Importantly, pharmacokinetic analysis revealed that following a single oral dose of EB (50 mg/kg)—which has previously shown anti-neuroinflammatory efficacy in murine models—the achieved peak plasma concentration (C_max_ > 1.2 µM) remained substantially below concentrations associated with hepatocyte toxicity in this in vitro system. These findings collectively suggest that EB exhibits a preliminary favorable therapeutic window with low hepatotoxic risk in the experimental settings. Additionally, the comparison between QSAR modeling and in vitro cellular testing results implies that computational predictions should be complemented with experimental validation.

To assess whether EA and EB undergo metabolic detoxification or activation, we compared the hepatocyte toxicity of these isomers with their hydrolysis, oxidation, and hydrolysis–oxidation metabolites using a HepG2-HLMs co-culture model. The results demonstrated a significant reduction in cytotoxicity following HLM incubation, supporting that EA and EB are likely converted into less toxic metabolites, primarily mediated by hepatic detoxification enzymes, such as carboxylesterases and cytochrome P450 enzymes. Furthermore, hydrolysis metabolites displayed lower hepatocyte toxicity than oxidation metabolites. Building upon these findings, both EA and EB exhibit inherently low hepatocyte toxicity, with their hydrolytic and oxidative metabolites with much lower cytotoxic potential. These findings suggest that the risk of hepatotoxicity caused by oral administration of EA and EB is likely to be low in vivo.

Notably, the HepG2-HLM co-culture model employed in this study has several limitations. First, the HepG2 cell model itself presents several inherent limitations, including limited metabolic capacity relative to primary human hepatocytes due to lower expression levels of phase I/II metabolic enzymes and transporters, as well as failure to recapitulate the complex in vivo hepatic microenvironment, particularly the absence of immune cells, which precludes modeling immune-mediated or idiosyncratic drug-induced liver injury mechanisms. Accordingly, the current results only reflect the direct cytotoxicity of EA/EB and their metabolites on hepatocytes and cannot represent the comprehensive liver injury effects in the in vivo immune microenvironment. Second, the present study only clarifies the overall detoxification trend of EA/EB metabolism but fails to explore the detailed molecular mechanisms underlying the lower cytotoxicity of hydrolytic/oxidative metabolites, and the mechanistic investigations will be performed in future studies. Third, the metabolites in this study were generated in situ by the HepG2-HLM co-culture system, forming metabolite mixtures rather than isolated monomeric metabolites. Thus, the current results only reflect the collective cytotoxic effect of each metabolite mixture, and the specific contribution of a single metabolite to metabolic detoxification needs to be further verified by preparing isolated metabolites in subsequent studies. Finally, in the present study, we only verified that EA/EB undergo metabolic detoxification via the hepatic hydrolysis pathway in the HepG2-HLM co-culture system. It should be clearly stated that these in vitro results merely reflect the regulatory effect of hepatic metabolism on compound toxicity, and cannot be directly inferred to indicate differences in in vivo safety between oral and parenteral administration. The actual safety of oral administration is also affected by multiple factors, including gastrointestinal absorption, the first-pass effect, and in vivo distribution. Further validation through in vivo animal experiments is required to compare the safety profiles of different administration routes.

## 5. Conclusions

Metabolic profiling using HLMs revealed 16 EA and 19 EB metabolites, and hydrolysis emerged as the primary metabolic pathway for both compounds. Both EA and EB demonstrated low hepatocyte toxicity in their parent forms. They undergo metabolic detoxification primarily through hydrolysis and oxidation, with hydrolysis metabolites being less toxic to hepatocytes than oxidative products.

## Figures and Tables

**Table 1:** Analysis of EA potential metabolites.

Peak ID	Retention Time (min)	Metabolic Process	Formula	Ion Mode	*m/z*	ppm	Peak Area	Score (%) *
M1	2.96	Hydrolysis + Ketone Formation	C_5_H_6_O_3_	-	113.0241	−2.5	885	73.6
M2	5.63	Ketone Formation	C_24_H_28_O_10_	-	475.1603	−1.4	9130	75.0
M3	6.56	Hydrolysis	C_5_H_8_O_3_	-	115.0400	−0.6	13,200	75.0
M4	6.56	Hydrolysis	C_20_H_26_O_7_	-	377.1601	−1.2	70,200	75.0
M5	6.77	Double bond reduction	C_24_H_32_O_9_	+	465.2085	−7.4	73,800	49.1
M6	6.81	Hydrolysis + Oxidation	C_17_H_22_O_7_	-	337.1290	−0.7	48,700	75.0
M7	6.90	Hydrolysis + Glucose Conjugation + Dehydroxylation	C_26_H_36_O_10_	+	509.2350	−6.2	45,100	39.6
M8	6.93	Hydrolysis	C_17_H_22_O_6_	-	321.1341	−1.0	121,000	75.0
M9	6.98	Hydrolysis + Dehydroxylation	C_20_H_26_O_5_	+	347.1821	−9.3	107,000	56.8
M10	7.07	Hydrolysis	C_22_H_28_O_8_	-	419.1714	0.6	240,000	75.1
M11	7.07	Hydroxylation	C_24_H_32_O_10_	-	479.1922	−0.2	135,000	75.0
M12	7.10	Hydrolysis + Oxidation	C_22_H_28_O_9_	-	435.1659	−0.4	27,500	75.0
M13	8.43	Dehydroxylation	C_23_H_28_O_8_	+	433.1840	−3.9	37,800	70.2
M14	8.57	Dehydroxylation + Deesterification	C_22_H_28_O_6_	+	389.1927	−8.1	24,600	59.8
M15	11.04	Hydrolysis + Oxidation	C_20_H_26_O_8_	+	395.1722	5.3	18,900	66.7
M16	11.77	Dehydroxylation + Deesterification	C_19_H_24_O_6_	+	349.1680	9.9	3170	42.7

* Score (%) indicates the confidence score for metabolite identification, reflecting the reliability of the software’s structural prediction/matching result for the metabolite (higher scores indicate more credible identification). Score (%) is a composite assessment based on multiple parameters, with core criteria including: (1) accuracy of mass match (ppm deviation); (2) degree of fragment ion matching (agreement between experimental and theoretical fragments); (3) plausibility of metabolic transformations (chemical feasibility of reactions such as hydrolysis or oxidation), and (4) retention time consistency. The specific algorithm follows a proprietary weighted scoring model, ultimately presented as a percentage (typically, a score ≥70% is considered a reasonably reliable identification) (https://sciex.com/content/dam/SCIEX/pdf/tech-notes/all/mass-spectrometry-BuspironePK-1270210.pdf).

**Table 2:** Analysis of EB potential metabolites.

Peak ID	Retention Time (min)	Metabolic Process	Formula	Ion Mode	*m/z*	ppm	Peak Area	Score (%) *
M1	2.21	Hydrolysis + Glutamine Conjugation	C_10_H_16_N_2_O_5_	-	243.0989	0.9	2940	50.0
M2	2.40	Hydrolysis + Glutamine Conjugation + Dehydroxylation	C_10_H_16_N_2_O_4_	+	229.1177	−2.8	33,000	48.1
M3	4.30	Hydrolysis + Glucose Conjugation	C_26_H_36_O_11_	+	525.2317	−2.6	17,400	36.1
M4	6.03	Double bond reduction	C_24_H_32_O_9_	+	465.2086	−7.0	25,100	62.4
M5	6.21	Deesterification + Demethylation to Carboxylic Acid	C_20_H_24_O_7_	+	377.1559	−9.6	27,200	56.0
M6	6.25	Hydrolysis	C_20_H_26_O_7_	-	377.1599	−1.7	27,000	75.0
M7	6.25	Hydrolysis + Hydroxylation	C_22_H_30_O_9_	-	437.1820	0.7	11,700	75.0
M8	6.88	Deesterification + Glucose Conjugation	C_26_H_36_O_10_	+	509.2339	−8.3	61,300	34.3
M9	6.96	Deesterification	C_20_H_26_O_5_	+	347.1819	−9.7	166,000	55.9
M10	7.03	Ketone Formation	C_24_H_28_O_10_	-	475.1603	−1.4	50,600	75.0
M11	7.46	Hydrolysis	C_22_H_28_O_8_	-	419.1711	−0.1	9700	75.0
M12	7.68	Hydrolysis + Oxidation	C_17_H_22_O_7_	-	337.1288	−1.3	39,400	75.0
M13	7.70	Hydrolysis	C_5_H_8_O_3_	-	115.0401	0.0	51,900	75.0
M14	7.72	Hydroxylation	C_24_H_32_O_10_	-	479.1918	−1.0	57,200	75.0
M15	7.75	Deesterification + Glucuronidation	C_23_H_30_O_11_	-	481.1711	−0.8	16,800	62.5
M16	7.77	Hydrolysis	C_17_H_22_O_6_	-	321.1341	−0.7	84,200	75.0
M17	8.01	Oxidation	C_24_H_30_O_10_	+	479.1900	−2.4	12,500	73.9
M18	8.53	Deesterification + Demethylation to Carboxylic Acid	C_17_H_20_O_6_	+	321.1304	−9.1	37,600	57.4
M19	8.92	Deesterification + Glucuronidation	C_26_H_34_O_11_	-	521.2034	1.1	13,100	50.0

* Score (%) indicates the confidence score for metabolite identification, reflecting the reliability of the software’s structural prediction/matching result for the metabolite (higher scores indicate more credible identification). Score (%) is a composite assessment based on multiple parameters, with core criteria including: (1) accuracy of mass match (ppm deviation); (2) degree of fragment ion matching (agreement between experimental and theoretical fragments); (3) plausibility of metabolic transformations (chemical feasibility of reactions such as hydrolysis or oxidation) and (4) retention time consistency. The specific algorithm follows a proprietary weighted scoring model, ultimately presented as a percentage (typically, a score ≥70% is considered a reasonably reliable identification) (https://sciex.com/content/dam/SCIEX/pdf/tech-notes/all/mass-spectrometry-BuspironePK-1270210.pdf).

**Table 3:** Toxicity prediction results of EA and EB by ADMET Predictor 8.5.

	Acute Toxicity ^1^		Hepatotoxicity ^2^	
EA	Rat_Acute	969.04	Ser_AST	Elevated
			Ser_ALT	Elevated
			Ser_LDH	Normal
	Prediction result	Negative	Prediction result	Negative
EB	Rat_Acute	634.11	Ser_AST	Elevated
			Ser_ALT	Elevated
			Ser_LDH	Normal
	Prediction result	Negative	Prediction result	Negative

^1^ The oral dose of ≥ [200, 320] mg/kg in rat acute toxicity studies was predicted to have low or negligible toxicity. ^2^ “Ser_AST = Elevated” AND “Ser_ALT = Elevated” AND “Ser_LDH = Elevated” were predicted to indicate hepatotoxicity. “Ser” refers to serum.

**Table 4:** Toxicity prediction results of EA and its metabolites by the DL-DILI Prediction Server.

ID	Formula	Phase I/II Metabolite	Metabolic Process	Prediction ^1^	Probability ^2^
EA	C_24_H_30_O_9_	—	—	Positive	0.6317
M1	C_5_H_6_O_3_	Phase I metabolite	Hydrolysis + Ketone Formation	Positive	0.6979
M2	C_24_H_28_O_10_		Ketone Formation	Positive	0.5855
M3	C_5_H_8_O_3_		Hydrolysis	Positive	0.5958
M4	C_20_H_26_O_7_		Hydrolysis	Positive	0.6357
M5	C_24_H_32_O_9_		Double bond reduction	Positive	0.6533
M6	C_17_H_22_O_7_		Hydrolysis + Oxidation	Positive	0.7919
M8	C_17_H_22_O_6_		Hydrolysis	Positive	0.8272
M9	C_20_H_26_O_5_		Hydrolysis + Dehydroxylation	Positive	0.8500
M10	C_22_H_28_O_8_		Hydrolysis	Positive	0.6365
M11	C_24_H_32_O_10_		Internal Hydrolysis	Positive	0.6128
M14	C_22_H_28_O_6_		Loss of O and C_2_H_2_O_2_	Positive	0.7183
M15	C_20_H_26_O_8_		Hydrolysis + Oxidation	Positive	0.6144
M16	C_19_H_24_O_6_		Loss of C_5_H_6_O_3_	Positive	0.6958
M12	C_22_H_28_O_9_		Hydrolysis + Oxidation	Negative	0.5364
M13	C_23_H_28_O_8_		Loss of C_19_H_22_O_6_	Negative	0.5796
M7	C_26_H_36_O_10_	Phase II metabolite	Hydrolysis + Glucose Conjugation	Negative	0.4853

^1^ Predicted label of the molecules. ^2^ Predicted probability for DILI-positive.

**Table 5:** Toxicity prediction results of EB and its metabolites by the DL-DILI Prediction Server.

ID	Formula	Phase I/II Metabolite	Metabolic Process	Prediction ^1^	Probability ^2^
EB	C_24_H_30_O_9_	—	—	Positive	0.7278
M4	C_24_H_32_O_9_	Phase I metabolite	Double bond reduction	Positive	0.8106
M5	C_20_H_24_O_7_		Loss of C_2_H_2_O_2_ and C_2_H_2_O_2_ + Demethylation to Carboxylic Acid	Positive	0.7201
M6	C_20_H_26_O_7_		Hydrolysis	Positive	0.7919
M9	C_20_H_26_O_5_		Loss of C_2_H_2_O_2_ and C_2_H_2_O_2_	Positive	0.8243
M10	C_24_H_28_O_10_		Ketone Formation	Positive	0.7226
M11	C_22_H_28_O_8_		Hydrolysis	Positive	0.7568
M12	C_17_H_22_O_7_		Hydrolysis + Oxidation	Positive	0.8165
M13	C_5_H_8_O_3_		Hydrolysis	Positive	0.8602
M14	C_24_H_32_O_10_		Internal Hydrolysis	Positive	0.5859
M16	C_17_H_22_O_6_		Hydrolysis	Positive	0.8969
M17	C_24_H_30_O_10_		Oxidation	Positive	0.7951
M7	C_22_H_30_O_9_		Hydrolysis + Internal Hydrolysis	Negative	0.5425
M18	C_17_H_20_O_6_		Loss of C_2_H_2_O_2_ and C_5_H_6_O_3_ + Demethylation to Carboxylic Acid	Negative	0.5812
M1	C_10_H_16_N_2_O_5_	Phase II metabolite	Loss of C_19_H_22_O_6_ + Glutamine Conjugation	Positive	0.5919
M3	C_26_H_36_O_11_		Hydrolysis + Glucose Conjugation	Positive	0.7691
M8	C_26_H_36_O_10_		Loss of C_2_H_2_O_2_ and C_2_H_2_O_2_ + Glucose Conjugation	Positive	1.0000
M15	C_23_H_30_O_11_		Loss of C_2_H_2_O_2_ and C_5_H_6_O_2_ + Glucuronidation	Positive	0.7366
M19	C_26_H_34_O_11_		Loss of C_2_H_2_O_2_ and C_2_H_2_O_2_ + Glucuronidation	Positive	1.0000
M2	C_10_H_16_N_2_O_4_		Loss of C_19_H_22_O_7_ + Glutamine Conjugation	Negative	0.5422

^1^ Predicted label of the molecules. ^2.^ Predicted probability for DILI-positive.

**Table 6:** The IC_50_ values of EA and EB in HepG2 cells after EA or EB exposure for 4 h with or without HLMs/NADPH/BNPP.

Treatment Group	Metabolic Process	IC_50_ [95% CI]/µM	
		EA	EB
EA/EB	—	101.9 [93.22, 114.2]	94.12 [81.30, 115.2]
EA/EB + HLMs + NADPH	oxidation–hydrolysis	Unable to calculate [very wide]	104.1 [very wide]
EA/EB + HLMs	hydrolysis	2063 [very wide]	111.2 [very wide]
EA/EB + HLMs + NADPH + BNPP	oxidation	103.9 [very wide]	101.2 [98.40, 104.9]

“Unable to calculate” indicates that the treatment did not reduce the viability of HepG2 cells to below 50% within the tested concentration range (0~100 μM). In other words, the IC_50_ value was greater than 100 μM, and an accurate IC_50_ value could not be obtained. The phrase “very wide” indicates that an accurate 95% confidence interval could not be determined for this treatment group within the tested concentration range (0~100 μM).

## Data Availability

The original contributions presented in this study are included in the article/Supplementary Materials. Further inquiries can be directed to the corresponding author.

## Supplementary Materials

The following supporting information can be downloaded at: https://www.mdpi.com/article/10.3390/toxics14030235/s1. Supplementary Material S1: Table S1. The major MS parameters for EA and EB metabolites detection; Figure S1. Characterization of parent EA. Representative extracted ion chromatogram, full-scan mass spectrum, and tandem mass spectra of EA, along with its proposed structure and fragmentation pathway; Figure S2. Characterization of parent EB. Representative extracted ion chromatogram, full-scan mass spectrum, and tandem mass spectra of EB, along with its proposed structure and fragmentation pathway; Figure S3. Characterization of EA-M10. Representative extracted ion chromatogram, full-scan mass spectrum, and tandem mass spectra of EA-M10, along with its proposed structure and fragmentation pathway; Figure S4. Characterization of EB-M9. Representative extracted ion chromatogram, full-scan mass spectrum, and tandem mass spectra of EB-M9, along with its proposed structure and fragmentation pathway; Figure S5. Dose–response curves and corresponding IC_50_ values for EA and EB in HepG2 cells under different incubation conditions. Supplementary Material S2: Policy Statements regarding human and animal tissues.
