# Supplementary material for: Metabolic Profiling and Detoxification of Eupalinolide A and B in Human Liver Microsomal Systems
Source: Toxics. 2026 Mar 9;14(3):235. doi: 10.3390/toxics14030235 (PMC13030334; doi:10.3390/toxics14030235)
Supplement: Supplementary file 1 [file toxics-14-00235-s001.zip › Supplementary material 1.pdf]

Supplementary Material 1

Table S1. The major MS parameters for EA and EB metabolites detection

	**Parameters**	**Ion mode**	
		+	−
MS	Ion Source Gas 1 (GS1)	60 psi	60 psi
	Ion Source Gas 2 (GS2)	60 psi	60 psi
	Curtain Gas (CUR)	35 psi	35 psi
	Temperature (TEM)	600 ℃	600 ℃
	IonSpray Vlotage Floating (ISVF)	5500 kV	-4500 kV
	TOF Masses (Da)	100~1000	100~1000
	Declustering Potential (DP)	80 V	-80 V
	Collision Energy (CE)	10 eV	-10 eV
MS/MS	Declustering Potential (DP)	80 V	-80 V
	Collision Energy (CE)	35 eV	-35 eV
	Collision Energy Spread (CES)	15	15
	TOF Masses (Da)	50~800	50~800

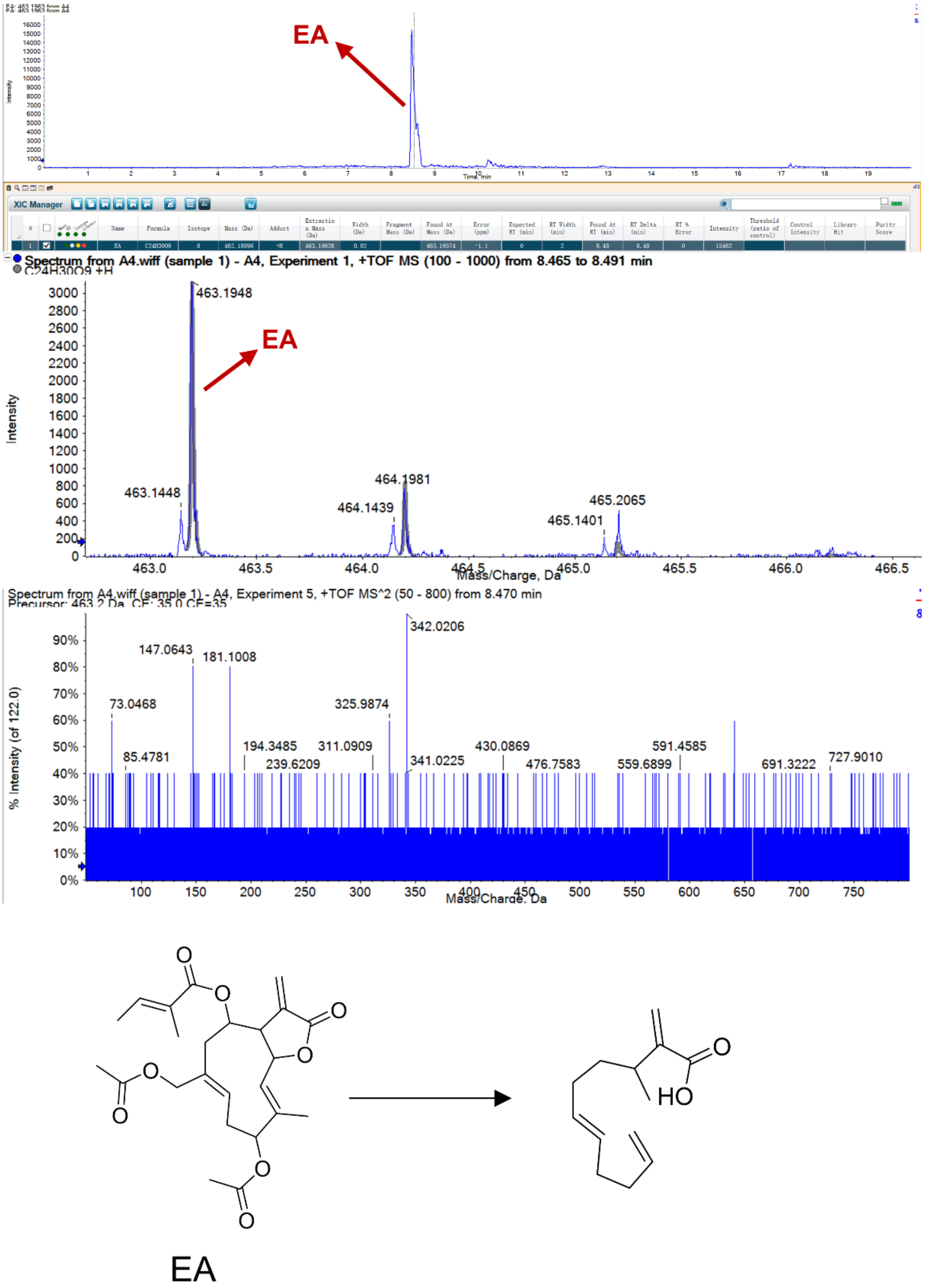

Figure S1. Characterization of EA. Representative extracted ion chromatogram, full-scan mass spectrum, and tandem mass spectra of EA, along with its proposed structure and fragmentation pathway.

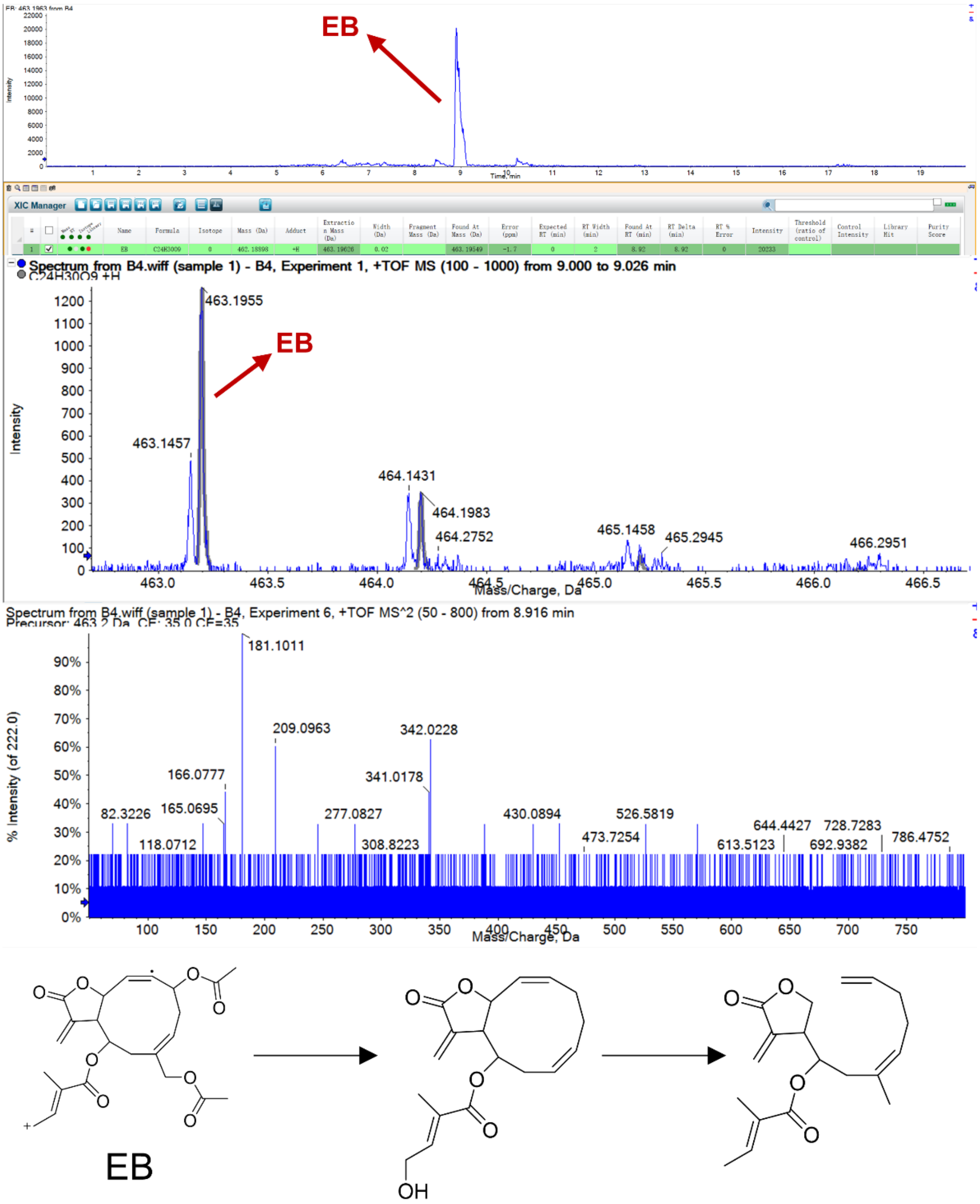

Figure S2. Characterization of EB. Representative extracted ion chromatogram, full-scan mass spectrum, and tandem mass spectra of EB, along with its proposed structure and fragmentation pathway.

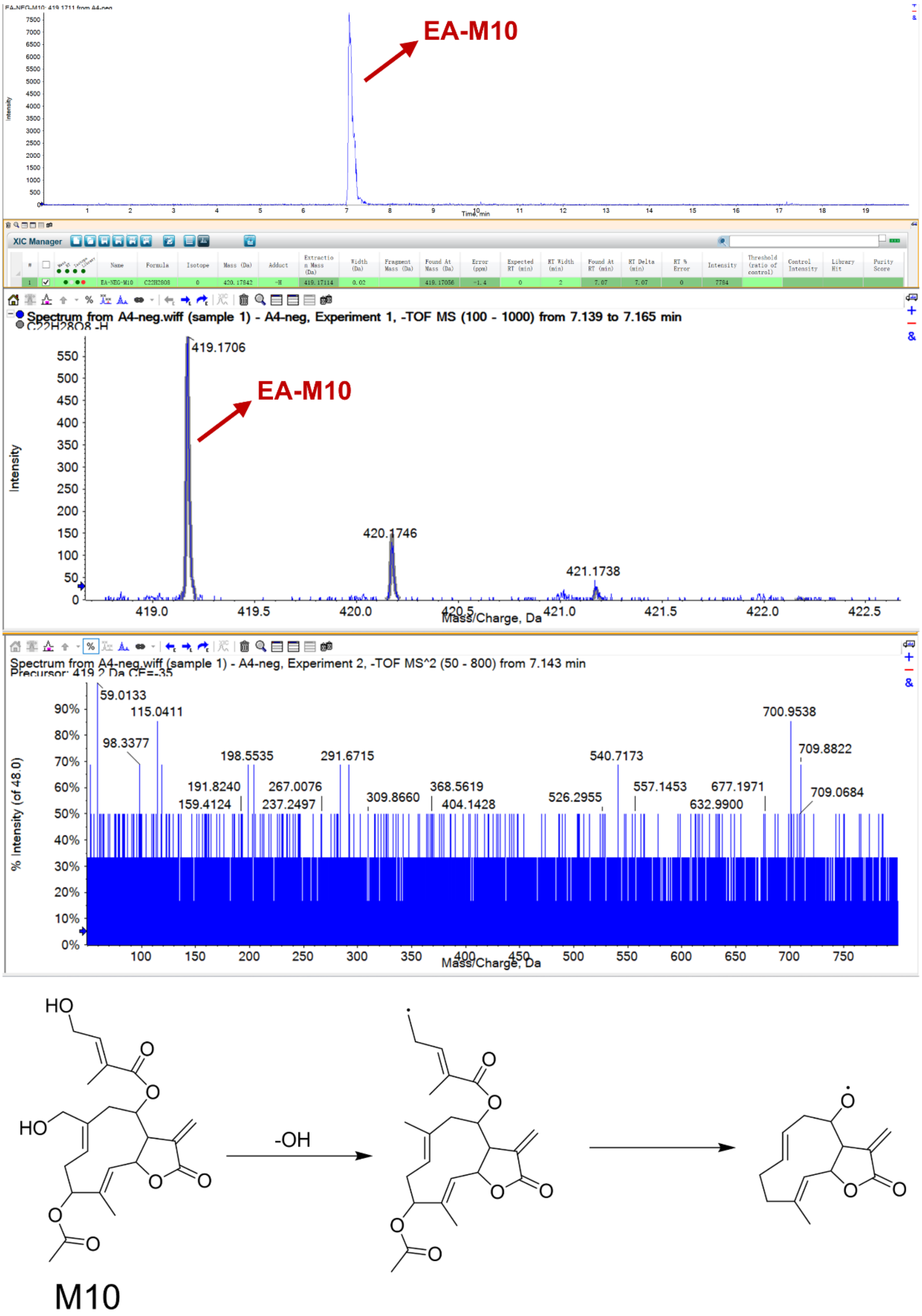

Figure S3. Characterization of EA-M10. Representative extracted ion chromatogram, full-scan mass spectrum, and tandem mass spectra of EA-M10, along with its proposed structure and fragmentation pathway.

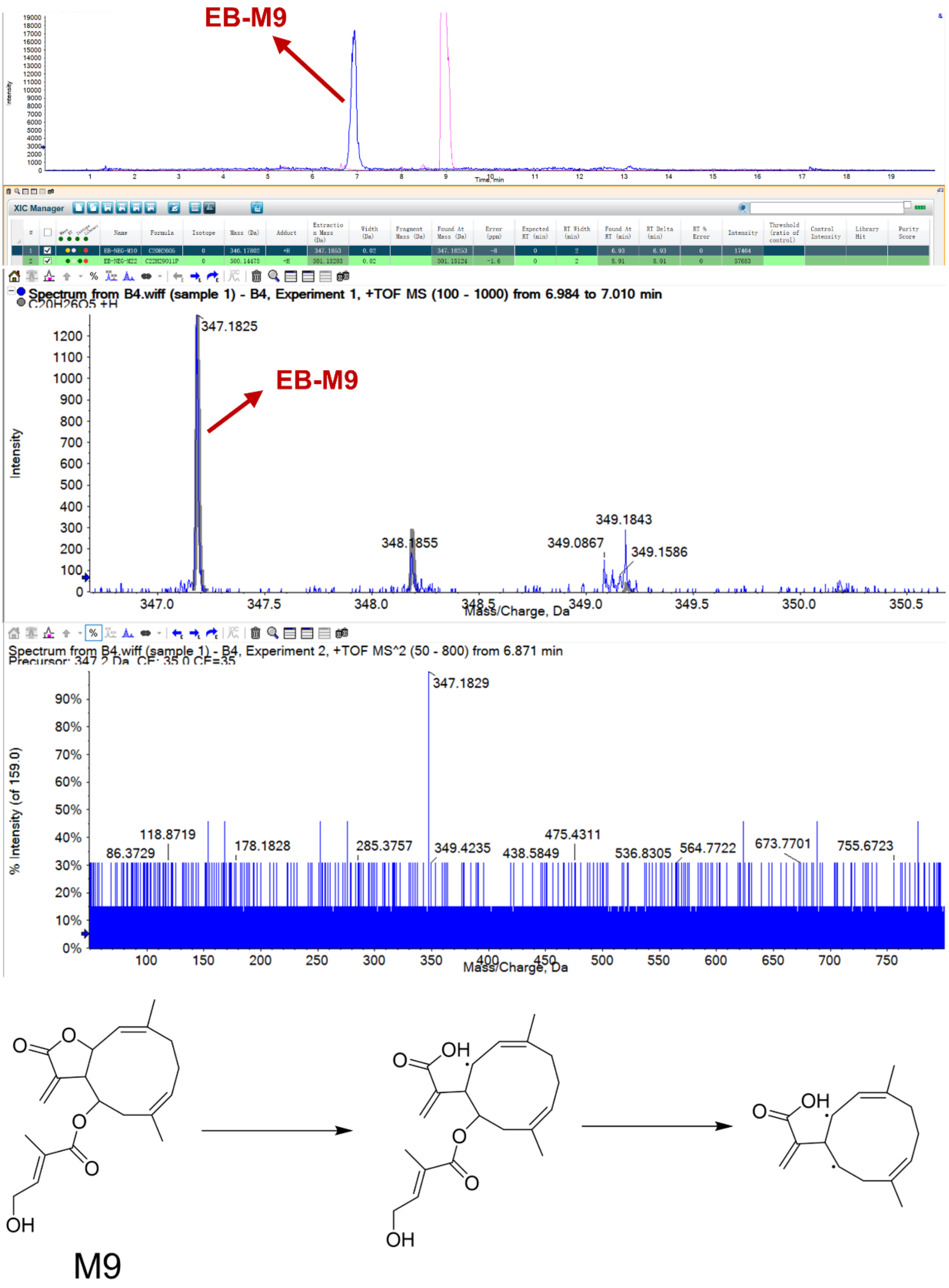

Figure S4. Characterization of EB-M9. Representative extracted ion chromatogram, full-scan mass spectrum, and tandem mass spectra of EB-M9, along with its proposed structure and fragmentation pathway.

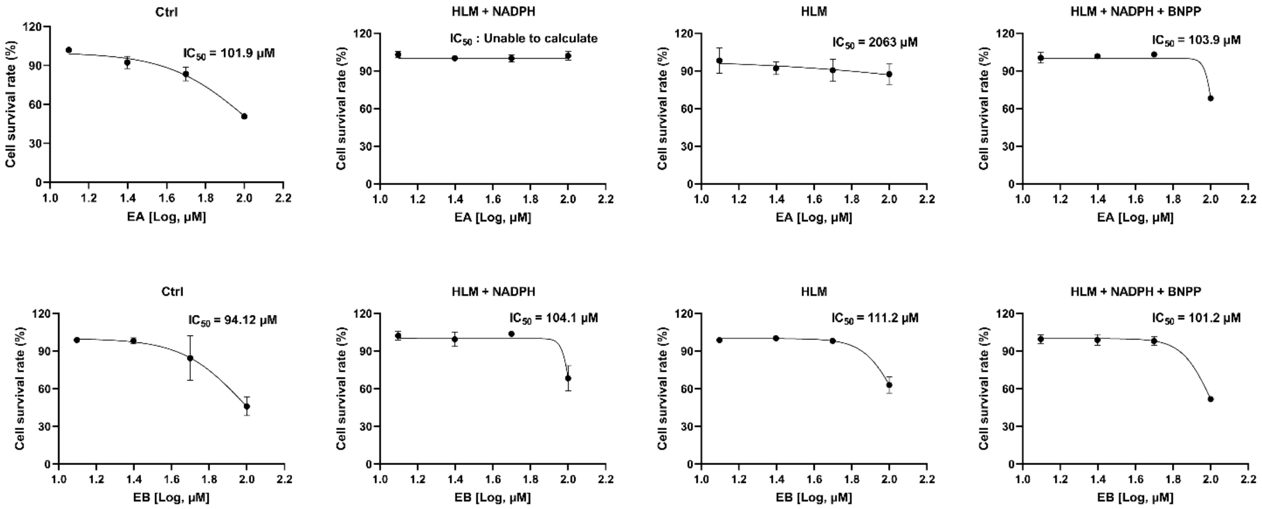

Figure S5. Dose-response curves and corresponding IC_{50} values for EA and EB in HepG2 cells under different incubation conditions.
