# Supplementary material for: Metabolic Profiling and Detoxification of Eupalinolide A and B in Human Liver Microsomal Systems
Source: Toxics. 2026 Mar 9;14(3):235. doi: 10.3390/toxics14030235 (PMC13030334; doi:10.3390/toxics14030235)
Supplement: Supplementary file 1 [file toxics-14-00235-s001.zip › Supplementary material 2.pdf]

Policy Statements regarding human and animal tissues

Human-derived biological material, and that from primates, should always be considered biohazardous (i.e., capable of harboring an infectious agent). Accordingly, when working with primate and human biological material, personnel should wear laboratory coats, eye protection and appropriate protective gloves.

1. Policy Statement Concerning Human Tissues

Sekisui XenoTech acquires human tissues and organs through partnerships with non-profit Organ Procurement Organizations (OPO). These partnerships are managed by Human Tissue and Organ Recovery and Placement Networks (HTORPN), which coordinate the distribution of human tissues/organs from OPOs to Sekisui XenoTech. Approved protocols by the Human Subjects Committee at the University of Kansas School of Medicine detail the use of these tissues. Sekisui XenoTech is exempt from further review due to the concealed identity of tissue donors and the public availability of the provided tissues. All human-derived samples acquired, processed, and distributed by Sekisui XenoTech are for research purposes only.

Donor Information

A single organization regulates and oversees the use of human tissue intended for transplantation in the United States, namely the United Network for Organ Sharing (UNOS). Patient confidentiality is protected by the policies and standards of UNOS and each respective OPO. Availability and release of patient information is subject to prevailing US Health Insurance Portability and Accountability Act of 1996 (HIPAA) regulations governing protected health information and patient privacy. HIPAA does not apply to the data exchange between the OPOs and HTORPNs due to the fact that OPOs are not covered entities. HIPAA does apply to the donor hospital. The HTORPNs with which Sekisui XenoTech contracts human tissue acquisition only provide anonymous and/or de-identified information and do not provide patient information that is considered Protected Health Information as defined in the HIPAA Act. Regulations in the United States require that, regardless of whether the organ is intended for transplantation or research purposes, the organ donor's identity be treated as highly confidential information. The OPOs generate an independent Donor Medical History and Interview Form and Donor Worksheet from the hospital medical records of each donor. From this OPO form, the HTORPNs create Donor Demographic Forms, which are completely anonymized and provided to Sekisui XenoTech.

Sekisui XenoTech does request the following donor information: sex, age, ethnicity, cause of death, stress levels of marker proteins (e.g., bilirubin, AST/ALT, etc.) disease status (e.g., diabetes, hypertension, etc.), medications, illicit drug use, alcohol use, smoking habits and serology information.

Informed consent statement regarding human tissues

Organ donors may elect to have their organs used either for transplantation only, or for a dual designation such as transplantation or research. Thus, the donor (or the donor's family) has the

1101 West Cambridge Circle Dr. │ Kansas City KS 66103 │ 913.438.7450 │ fax 913.227.7171 │ xenotech.com

right to prevent the use of the donor's organs for research. Regardless of the use of donated organs, no compensation is given to the donor's family or accomplices; any such compensation is illegal in the United States. In those cases where donors (or family members) elect to withhold organs from research uses, any organs that cannot be transplanted are properly discarded.

It is the hospital and OPO's intentions that tissues be used for transplant, but if a recipient is not found, or the surgeon refuses the tissue for specific reasons, then the tissues may become available for research purposes. Consent for research is obtained by each individual Organ Procurement Organization and the information included in each consent form is the sole discretion of that OPO in compliance with local, state and federal guidelines including the State Uniform Anatomical Gift Act. Each consent form includes language that indicates that the donated tissue can be used for research and is an anatomical gift for which no compensation is given. Sekisui XenoTech receives hepatic, renal, intestinal, pulmonary, and other human tissue from various regional OPOs that obtain organs approved for research use. Organ procurement organizations maintain the informed consent records from each donor, and it is the policy of each HTORPN to confirm the existence of informed consent for research purposes prior to transport of organs to Sekisui XenoTech. This procedure is intended to ensure that Sekisui XenoTech manufactures human-derived products only when informed consent has been granted for research use of those specific organs. Sekisui XenoTech does not, and, in consideration of confidentiality, cannot obtain the informed consent records from OPOs or HTORPNs, nor does Sekisui XenoTech provide originals or copies of documents obtained through human tissue procurement in reported data or contract study materials.

Sekisui XenoTech does not deal directly with - nor does it make any direct payments to - the surgeons who procure organs or the medical institutions where they work.

Organ Procurement Organizations

A single organization regulates and oversees the use of human tissue intended for transplantation in the United States, namely the United Network for Organ Sharing (UNOS). All OPOs must be certified by the Centers for Medicare and Medicaid services (CMS) and abide by CMS regulations. By federal law, all OPOs must be members of the Organ Procurement and Transplantation Network (OPTN) and, as such, are members of the United Network for Organ Sharing (UNOS) which is a privately operated entity that has a contract with the U.S. government to operate the OPTN. These OPOs operate under a set of standards established by the Association of Organ Procurement Organizations (AOPO) and UNOS. These OPOs refer non-transplantable organs and tissues for placement with medical research where proper authorization for medical research has been obtained and which is documented on each OPO's Authorization Form. Each OPO operates individually under its own protocol to obtain authorization. These standard operating procedures must conform to each respective state's Uniform Anatomical Gift Act (UAGA) as well as the hospitals' policies in each region.

Every two years the OPOs must reapply for membership to UNOS. To date, all OPOs remain in good standing as members of UNOS. As a member, the OPOs agree to comply with all applicable provisions of the National Organ Transplant Act (NOTA), as amended; OPTN Final Rule, 42 CFR Part 121; UNOS Bylaws and Policies as in effect from time to time. Additionally, every three years, OPOs volunteer to become accredited by AOPO whose policies for ethical practices in donation must be complied. All of the specimens distributed through HTORPNs are obtained from such OPOs, all of whom are non-profit organizations who are legally mandated to

obtain authorization from the legal next-of-kin in accordance with each state's UAGA, or abide by First Person Consent as documented in a Donor Registry.

Expectations of Tissue Suppliers and Their Ethics Policies/Practices

It is Sekisui XenoTech's understanding that the HTORPNs, from which human tissues are distributed for research purposes, are in good standing with the Organ Procurement and Transplantation Network (OPTN) and the National Organ Transplant Act, as amended. Additionally, Sekisui XenoTech also expects that these organizations will maintain documentation of informed consent from the donor's family.

Sekisui XenoTech requalifies HTORPNs as human tissue providers every three years and requests information regarding the policies that they have in place and the regulations which they follow. Sekisui XenoTech does not require ethics documentation from HTORPNs because they publicly provide this information on their websites.

Serologies

All human tissues accepted by Sekisui XenoTech have been tested for the possible presence of various infectious diseases, and Sekisui XenoTech does not accept human-derived material unless the donor has tested negative (non-reactive) for RPR, HIVAb, HBsAg, HCVAb, and SARS-CoV-2 (as of March 2020). All human tissue is also tested for CMVAb. However, due to the widespread (nearly ubiquitous) appearance of CMV in the population, and its relative insignificance as an infectious agent, tissues from donors reactive for CMVAb are accepted. The serology status of each donor is typically determined by ELISA or Nucleic Acid Test by the organ procurement hospital.

Human Tissue Handling, Storage, and Identification

Sekisui XenoTech is an access-controlled facility and all human tissues and derivatives are stored under appropriate conditions (≤-80°C) in locked storage units with limited access.

Human tissues received at Sekisui XenoTech are tested and verified to be negative for known bloodborne pathogens and are not procured from high-risk donors. As such, none of the human tissues utilized at Sekisui XenoTech are reasonably expected to be infectious. Although human tissues received at Sekisui XenoTech are not known to be hazardous, fresh human tissues are treated as potentially biohazardous. It is recommended that processed human-derived samples are handled with the same PPE and caution as biohazardous materials, even though they pose little biohazardous risk.

Human tissues are typically received via a courier service from a donating hospital or organ procurement agency. Upon receipt, the shipping container is visually inspected for signs of structural damage or leakage. Contents are inspected to ensure sufficient wet ice, cold packs, or dry ice is included to adequately keep the tissue chilled during shipment.

Each tissue sample received at Sekisui XenoTech is assigned a tissue identification number. This number is unique to each tissue received from each donor and is used to track the tissue

1101 West Cambridge Circle Dr. │ Kansas City KS 66103 │ 913.438.7450 │ fax 913.227.7171 │ xenotech.com

throughout the processing procedure. Each unique product lot number and tissue identification number can be used to track the donor(s) comprising each cellular or subcellular product.

Fetal Tissues

As a matter of corporate policy, Sekisui XenoTech does not accept tissues from aborted fetuses, regardless of whether the fetus was spontaneously or intentionally aborted.

ICH-GCP Regulations and the Declaration of Helsinki

Sekisui XenoTech's procedures involve the use of human-derived materials only. Therefore, Sekisui XenoTech is exempt from these policies. Sekisui XenoTech's procedures do not involve studies with human subjects, only with human-derived materials obtained with appropriate informed consent.

Federal Policy for the Protection of Human Subjects in Research

The Federal Policy for the Protection of Human Subjects in Research, known as the "Common Rule" ("45CFR46") spells out the requirements for conducting research involving human subjects and the need for research proposals to be reviewed by an Institutional Review Board (IRB), along with elements of informed consent.

Since the donors of non-transplantable organs are deceased and their personally identifiable information is not made available by HTORPNs to Sekisui XenoTech, the Common Rule treats this research as not involving human subjects, and is therefore classified as exempt.

Acquisition of Tissues from Outside the U.S.

Sekisui XenoTech does not acquire tissues from other countries; therefore, laws from other countries do not impact Sekisui XenoTech's tissue acquisition process.

2. Policy statement concerning the safety of non-human primate-derived tissues

Sekisui XenoTech manufactures products derived from non-human primate organs for the purpose of xenobiotic research. All organs used to manufacture these products are obtained from reliable sources within the United States. All non-human primate organs used for product manufacture are obtained from disease-free (asymptomatic) animals under the care of a veterinarian.

In accordance with Federal (USDA) regulations, all non-human primates entering the United States are placed in quarantine for a period of thirty days. If an animal were infected with Ebola virus, it would die during the quarantine period, and hence, would not reside in a primate colony in the United States. For this reason, Ebola virus testing is not a common procedure in the United States. Animals are tested for Simian Herpes B virus (SBV), Simian Retrovirus (SRV), Simian Immunodeficiency virus (SIV), and Simian T-Lymphotropic Virus 1 (STLV-1). None of Sekisui XenoTech's animals examined for these pathogens tested positive.

1101 West Cambridge Circle Dr. │ Kansas City KS 66103 │ 913.438.7450 │ fax 913.227.7171 │ xenotech.com

Although animals are quarantined and tested for certain infectious diseases, it is not scientifically possible to guarantee that non-human primate-derived material is free of **all** infectious agents and poses absolutely no health risk. Therefore, we strongly caution all researchers who use non-human primate-derived material to treat such material as a potential biohazard, and to observe all national, regional, and local regulations governing the handling of such material. Even if non-human primate-derived material has been tested for the presence of specific infectious agents by highly sensitive techniques, such as RT-PCR, and found to test negative, the material should still be treated as a potential biohazard.

3. Policy statement concerning the safety of non-primate tissues

Sekisui XenoTech manufactures products derived from non-primate (e.g., dog, rabbit, rat, mouse, guinea pig, hamster, etc.) organs for the purpose of xenobiotic research. All organs used to manufacture these products are obtained from AAALAC (Association for Assessment and Accreditation of Laboratory Animal Care)-accredited facilities, which are registered as research facilities with the USDA-APHIS-AC (U.S. Department of Agriculture - Animal and Plant Health Inspection Service - Animal Care). All non-primate organs used for product manufacture are obtained from disease-free (asymptomatic) animals under the care of a veterinarian.

4. Policy statement regarding the ethical use of animals in research

An independent organization, specifically the Institutional Animal Care and Use Committee at the University of Kansas Medical Center, has reviewed and approved Sekisui XenoTech's protocol for the isolation of cells and subcellular fractions from various species to be used for research, including, but not limited to, drug metabolism and enzyme induction/inhibition studies. This committee audits Sekisui XenoTech twice in a calendar year to ensure that all federal and local policies and regulations, including those set forth by the USDA-APHIS-AC and AAALAC, are followed for the use of animals in research. Sekisui XenoTech is committed to the ethical treatment of animals in research and provides tissue-derived fractions in order to considerably decrease the number of animals required in traditional metabolism studies.

07 January 2022

Zell Woodworth
Division Director - Products

1101 West Cambridge Circle Dr. │ Kansas City KS 66103 │ 913.438.7450 │ fax 913.227.7171 │ xenotech.com
